# Myocardial Injury After Non-Cardiac Surgery in Otolaryngology: Evidence Gaps and a Systematic Review

**DOI:** 10.3390/jcm15062186

**Published:** 2026-03-13

**Authors:** Justyna Domka, Robert Kasza, Lidia Ziętek, Wiktoria Smyła-Gruca, Marta Antkowiak, Anna Koniewska, Marta Gamrot-Wrzoł, Denis Kowalski, Hanna Misiołek, Maciej Misiołek, Szymon Białka

**Affiliations:** 1 Clinical Department of Anaesthesiology and Intensive Care, University Clinical Hospital Fryderyk Chopin in Rzeszow, 35-055 Rzeszow, Poland; 2 Student Scientific Society of Anesthesiology and Intensive Care, Department of Anesthesiology and Intensive Care, Medical University of Silesia, 41-800 Zabrze, Poland; 3 Department of Otorhinolaryngology and Laryngological Oncology in Zabrze, Medical University of Silesia, 41-800 Zabrze, Poland; 4 Department of Anesthesiology and Intensive Care, Medical University of Silesia, 41-800 Zabrze, Poland

**Keywords:** ENT, MINS, otorhinolaryngology

## Abstract

**Background/Objectives***:* Myocardial injury after non-cardiac surgery (MINS) is a common and serious postoperative complication. Its largely asymptomatic course hampers early recognition, highlighting the importance of systematic biomarker monitoring. The aim of this review is to summarize current evidence on the diagnosis, risk factors, and management of MINS, with a focus on otolaryngology, where intraoperative hypotensive techniques may increase risk. **Methods:** A basic science review was conducted using PubMed, Embase, and the Cochrane Library (2005–2025). From 2712 records, 30 studies met the inclusion criteria after removing duplicates, screening titles/abstracts, and full-text assessment. These studies formed the basis for the final analysis. **Results**: Observational studies and reviews identify perioperative troponin monitoring as the diagnostic gold standard. However, no evidence-based management guidelines exist, and otorhinolaryngology-specific data remain rare but not entirely absent. Troponin elevation in the early postoperative period reliably predicts adverse outcomes. While MINS is well documented in vascular and orthopedic surgery, evidence in otolaryngology is limited. Controlled hypotension in procedures such as functional endoscopic sinus surgery or head and neck tumor resection may further elevate risk. **Conclusions**: MINS is an underrecognized complication with major prognostic significance. The lack of standardized management and the absence of large otolaryngology cohorts underscore an urgent need for targeted research and specialty-specific guidelines and support the justification for integrating existing evidence into otolaryngologic practice.

## 1. Introduction

A myocardial infarction (MI) is defined as a rise and/or fall in cardiac troponin with at least one value above the 99th percentile, accompanied by symptoms or objective signs of ischemia [1]. A broader concept encompassing asymptomatic myocardial damage is myocardial injury after non-cardiac surgery (MINS), defined as an elevation in cardiac necrosis biomarkers within 30 days postoperatively, regardless of clinical symptoms [2]. The condition is often underestimated due to the lack of routine troponin testing and its nonspecific presentation, yet it poses a serious risk to surgical patients.

Head and neck surgeries require particular attention because of rich vascularization and the widespread use of controlled hypotension to reduce bleeding and optimize visualization. Essential in procedures like functional endoscopic sinus surgery (FESS) or middle ear microsurgery, this strategy may increase myocardial ischemia and predispose to MINS [3,4]. Furthermore, many otorhinolaryngology patients are older, often with multiple comorbidities and a high baseline cardiovascular risk, which further increases the potential for perioperative myocardial injury.

Epidemiological data show that patients over 45 years of age are at the highest risk, with a frequency of cardiovascular events up to 25% following major vascular, orthopedic, or oncological operations [5,6]. The primary mechanism is perioperative myocardial ischemia, influenced by age, comorbidities, blood loss, hypotension, and tachycardia [7,8]. Similar factors are critical in otorhinolaryngology (ENT), where intraoperative hemodynamic management is often adjusted.

This review summarizes current knowledge on MINS, with a particular focus on ENT procedures and the role of controlled hypotension in its development [9].

## 2. Materials and Methods

This Systematic Review was conducted in accordance with PRISMA 2020 guidelines. The review protocol was not registered prospectively; however, detailed instructions for its completion were provided. The literature search and study selection process were conducted using a structured, multi-stage method. Six authors were involved in data curation, and four authors contributed to resource identification and database searching. The search process was conducted by using PubMed, Embase, and the Cochrane Library. Each database was searched independently using predefined search strategies. The following keywords and their combinations were applied using Boolean operators (AND/OR): (“myocardial injury after non-cardiac surgery” OR “MINS”) AND (“otorhinolaryngology” OR “ENT”). Where applicable, Medical Subject Headings (MeSH) and database-specific indexing terms were also used. The search was restricted to studies published between 2005 and 2025 and limited to the following article types: Clinical Study, Clinical Trial, Meta-Analysis, Randomized Controlled Trial, Review, and Systematic Review.

The initial database search identified 2712 records (PubMed *n* = 556, Embase *n* = 504, Cochrane *n* = 1652). After removing 2078 duplicates, 634 records remained for screening. Title and abstract assessment led to the exclusion of 556 articles that did not meet the predefined criteria. Full texts of 78 reports were sought, of which 11 could not be obtained.

A total of 67 full-text articles were assessed for eligibility. Full-text evaluation was performed independently by members of the review team, with final inclusion decisions reached by consensus. Following detailed evaluation, 39 were excluded for reasons such as inadequate outcome reporting, irrelevant study population, or lack of perioperative troponin assessment. Additionally, one relevant article was identified through citation searching and was included after full-text assessment.

**Figure 1:**
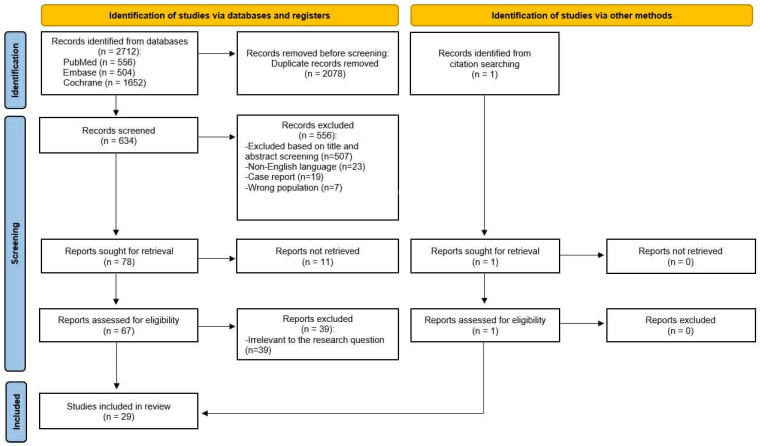
PRISMA flowchart of the medical database search strategy (Supplementary Materials).

In the end, 29 studies were included in the review and served as the basis for further analysis (Figure 1).

## 3. Results

### 3.1. Epidemiology and Diagnosis of MINS

Globally, over 300 million major non-cardiac surgeries are performed annually, with more than 1 million patients dying within 30 days [10]. Among individuals over 45 years, mortality is around 1% [11]. The incidence of MINS is estimated at 18%, making it one of the most common and clinically significant perioperative complications [12]. MINS is particularly dangerous because it is often clinically silent yet strongly predicts adverse outcomes. Postoperative troponin elevation is associated with more than a threefold increase in 30-day mortality and up to a sixfold increase in one-year mortality [13]. Large cohort studies confirm these findings, showing nearly a twentyfold increase in early postoperative mortality, a sevenfold increase within 30 days, and almost a fourfold increase within one year in patients with MINS compared to those without the condition [14].

Diagnosis relies on high-sensitivity cardiac troponins (hs-cTn T/I), which outperform conventional assays [14,15]. Comparing pre- and postoperative levels is essential, as more than half of surgical patients may already have elevated baseline troponins [15]. Interpretation is especially challenging in heart failure, renal dysfunction, sepsis, or systemic inflammation [1,8,16]. Routine troponin monitoring is rarely performed in many surgical fields, including ENT surgery; hence, the majority of MINS cases in this field are likely underrecognized. Prospective studies show that systematic hs-cTn testing detects MINS in approximately 16% of patients, while only 6–18% present clinical signs of ischemia [17]. Without routine monitoring, nearly 70% of cases may remain undiagnosed [18]. Data from the VISION study indicate the highest risk within 24–72 h postoperatively, supporting serial testing during this period [2,14,19].

Other diagnostic methods are of limited value: more than 80% of MINS cases show no new ECG changes [14], echocardiography detects wall motion abnormalities in only up to 30% [7,8], and among alternative biomarkers, only NT-proBNP has confirmed prognostic significance [5].

### 3.2. Pathophysiology

In approximately 86–89% of cases, the primary mechanism of MINS is myocardial ischemia [7]. Major risk factors include advanced age, coronary artery disease, chronic kidney disease, anemia, and intraoperative hypotension [15]. Two main pathophysiological types are distinguished:

- Type I—Acute coronary syndrome caused by rupture of an unstable atherosclerotic plaque with coronary obstruction. Perioperative hypertension, tachycardia, and stress-related neurohormonal disturbances further promote ischemia and troponin elevation [20];
- Type II—More common, resulting from an imbalance between myocardial oxygen supply and demand. Tachycardia, hypotension, anemia, or hypoxemia reduce coronary perfusion and precipitate ischemic episodes, especially in patients with chronic coronary artery disease [20].

**Figure 2:**
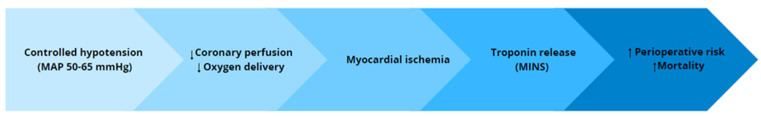
Mechanism of MINS in Otorhinolaryngology. Schematic representation of the pathophysiological cascade leading to myocardial injury after non-cardiac surgery in otolaryngology. Controlled hypotension during procedures such as functional endoscopic sinus surgery or middle ear microsurgery decreases mean arterial pressure, reduces coronary perfusion and oxygen delivery, and predisposes to myocardial ischemia, troponin release, and increased perioperative mortality. **Abbreviations**: MAP—mean arterial pressure; MINS—myocardial injury after non-cardiac surgery; ↑—increase; ↓—decrease.

Less frequent causes include sepsis, rapid atrial fibrillation, or pulmonary embolism, which can also elevate troponins and worsen prognosis [7]. The Mechanism of MINS in Otorhinolaryngology is shown in Figure 2.

### 3.3. MINS in Otorhinolaryngology

Otorhinolaryngological surgery is a clinically relevant setting in the context of perioperative myocardial damage. Although the literature on this topic is limited, and most of it relates to the general surgical patient population, the specific intraoperative conditions in head and neck surgery create a unique risk of developing myocardial ischemia.

### 3.4. Controlled Hypotension in FESS and Middle Ear Microsurgery—Necessity vs. Risk

Controlled hypotension is a cornerstone of anesthetic management in otorhinolaryngology, particularly in procedures concerning richly vascularized areas, such as functional endoscopic sinus surgery or middle ear microsurgery. Reducing mean arterial pressure (MAP) to 50–75 mmHg improves visibility and decreases intraoperative bleeding [21,22]. However, this comes at a cardiovascular cost: maintaining MAP below 55 mmHg for more than 1 min significantly increases the risk of myocardial injury, and each additional 10-min episode of hypotension raises this risk by approximately 30% [18,23,24].

The VISION substudy on major head and neck surgery confirmed the clinical relevance of this mechanism. Most first-detected cases of MINS occurred on postoperative day 2 (median onset: 2 days, range 1–23). Among the 648 patients included, nearly 70% of events were clinically silent and identified only by means of serial troponin testing. Given that controlled hypotension is frequently used in otorhinolaryngology, it likely represents a key perioperative factor predisposing to silent MINS in this group [18].

Pharmacological methods to achieve hypotension vary in their cardiovascular safety profiles. β-blockers and inhaled anesthetics provide relatively stable hemodynamics compared to sodium nitroprusside [25]. Dexmedetomidine may create optimal surgical conditions, but it carries a higher risk of bradycardia and profound hypotension, which could further predispose to MINS [21,22]. These findings suggest the need for judicious agent selection and strict time limitations on hypotension episodes to balance surgical needs with cardiac safety.

### 3.5. Head and Neck Cancer Surgery—Comorbidities and Perioperative Risk

Patients undergoing major head and neck surgery frequently present with significant comorbidities such as coronary artery disease, hypertension, diabetes, chronic kidney disease, or heart failure, which substantially increase the risk of perioperative myocardial injury. In the VISION substudy, the study population consisted predominantly of older adults: 58% aged 45–64 years, 25.8% aged 65–74 years, and 16.2% aged ≥75 years. The incidence of MINS rose accordingly, from 11.9% overall to 23.8% in patients aged ≥75 years.

MINS was more common in patients with comorbidities. Those who developed MINS were more likely to have a history of coronary artery disease, hypertension, diabetes, chronic heart failure, renal insufficiency, and tobacco use (median smoking history of 40 years). In this cohort, MINS was associated with a fivefold higher risk of 30-day mortality (HR 5.51; 95% CI, 1.75–17.36) and prolonged length of hospital stay when accompanied by ischemic features (+3.15 days; 95% CI, 1.47–6.76) [18].

Similar results were observed in a prospective analysis of 378 patients undergoing major head and neck cancer surgery, where postoperative troponin I elevation occurred in 15% of cases. Risk factors included chronic renal failure, coronary and peripheral vascular disease, hypertension, and prior combined radio-chemotherapy. Elevated troponin was associated with an eightfold increase in 60-day mortality, prolonged Intensive Care Unit (ICU) stay, longer hospitalization, and almost doubled one-year mortality [19].

These findings demonstrate that patients undergoing major head and neck procedures are typically older, multimorbid, and therefore highly predisposed to MINS. This reinforces the need for systematic troponin monitoring and close interdisciplinary management in this high-risk surgical population.

## 4. Discussion

### 4.1. Evidence Gap—Lack of Large Prospective ENT-Specific Cohorts

Despite increasing recognition of MINS, evidence in otorhinolaryngology remains scarce. More than 80% of cases are clinically silent, and because routine troponin testing is rarely performed in ENT departments, many complications go undetected [14]. Currently, there are no otorhinolaryngology-specific guidelines for postoperative troponin monitoring or overall MINS management, and most available data come from subgroup analyses of broader surgical cohorts, which may not reflect the unique hemodynamic challenges of head and neck procedures. Compared to vascular and orthopedic surgery, no large dedicated ENT-specific prospective cohort exists.

Large multicenter studies dedicated to ENT are urgently needed to establish the true incidence and prognostic impact of MINS, determine optimal hs-cTn monitoring strategies, and assess the influence of controlled hypotension and pharmacological interventions on outcomes.

**Figure 3:**
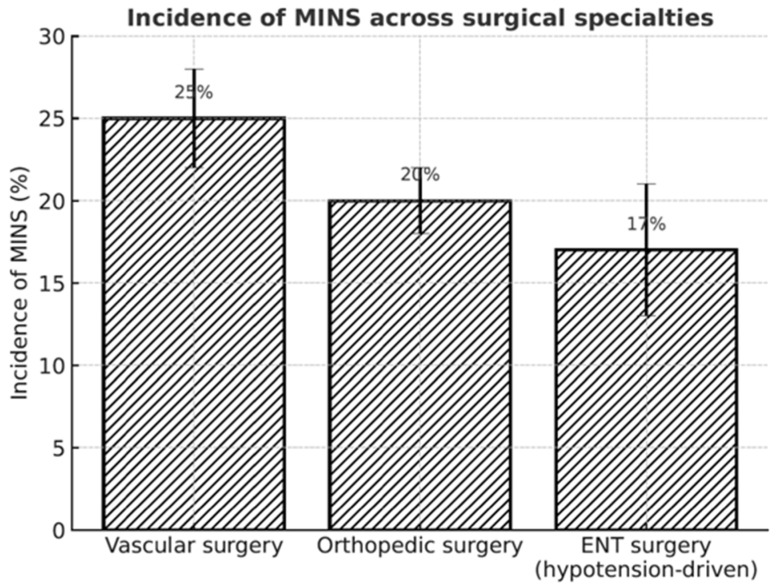
Incidence of MINS Across Surgical Specialities. Comparison of the reported incidence of MINS in major surgical fields. Vascular and orthopedic surgery cohorts show higher and consistently documented risk [11], while evidence in otolaryngology remains limited, primarily based on smaller prospective studies [18]. Error bars represent variability across reported studies. **Abbreviations**: MINS—myocardial injury after non-cardiac surgery; ENT—ear, nose, and throat.

From a practical point of view, it is necessary to limit the duration and depth of controlled hypotension to an absolute minimum, ensure close cooperation between surgeon and anesthesiologist in maintaining hemodynamic stability, and consider troponin monitoring in patients at risk (≥45 years of age, cardiovascular disease, chronic renal failure), even after procedures previously considered as minimally invasive [18,25]. Incidence of MINS Across Surgical Specialities is shown in Figure 3.

### 4.2. Management and Treatment Strategies

There is currently no clear guideline for the treatment of perioperative myocardial injury. Management is primarily based on prevention and modification of risk factors in the perioperative period, as well as appropriate cardiological supervision in the event of elevated cardiac markers [8].

### 4.3. Antiplatelet Therapy 

The most commonly used drug for the prevention of cardiovascular disease is acetylsalicylic acid (ASA). However, in the large POISE-2 study of more than 10,000 patients, ASA did not reduce the rate of myocardial infarction (6.2% vs. 6.3% in the placebo group) or the combined risk of death and myocardial infarction (7.0% vs. 7.1%), while it significantly increased the risk of major bleeding (4.6% vs. 3.8%) [26]. These results highlight the need to individualize the decision to use ASA in perioperative patients.

### 4.4. Control of Heart Rate and Blood Pressure

In practice, β-blockers and ivabradine are also used to reduce heart rate, but their use in MINS prevention is controversial. In the PREVENT-MINS study involving over 2000 patients, ivabradine effectively reduced heart rate but did not reduce the incidence of MINS (17.0% vs. 15.1% in the placebo group; RR 1.12; 95% CI 0.92–1.37) [27]. In the POISE-1 study, metoprolol reduced the incidence of heart attacks (4.2% vs. 5.7%), but at the cost of increased mortality (3.1% vs. 2.3%) and risk of stroke (1.0% vs. 0.5%) [28]. These data indicate that pharmacological control of hemodynamics may have the opposite effect to that intended and should be used carefully [8,12].

### 4.5. Statins

Unlike the above therapies, the use of statins has clear benefits. In a prospective analysis by Kashlan et al. involving over 2800 patients, statin use was associated with a 23% reduction in cardiac complications (8.6% vs. 11.2%) and a trend towards lower 30-day mortality (1.2% vs. 1.9%) [10]. Furthermore, in patients diagnosed with MINS, intensive statin therapy may reduce 30-day mortality by as much as 30–40% [8]. The greatest benefits are observed with high doses (atorvastatin 40–80 mg/day, rosuvastatin 20–40 mg/day), in compliance with the concepts of secondary prevention of coronary artery disease.

### 4.6. Anticoagulant Treatment

The strongest therapeutic evidence comes from the MANAGE study, in which the use of dabigatran (110 mg twice daily) in patients with MINS reduced the risk of major vascular complications (heart attack, stroke, venous thrombosis) by approximately 25–28% without significantly increasing major bleeding [29]. These results indicate that oral anticoagulant therapy may be a promising therapeutic strategy.

Evidence for MINS management strategies are summarized in Table 1.

### 4.7. Prospects and Challenges

Perioperative MINS is a significant clinical problem, the importance of which will grow with the increase in the number of non-cardiac surgical procedures performed, including those in the field of ENT. Up to 80% of MINS cases remain asymptomatic, leading to frequent underdiagnosis [1,2]. Despite the availability of high-sensitivity troponin assays, routine monitoring is still uncommon in ENT settings. Research data clearly indicate that serial hs-cTn testing in the first 24–72 h after surgery allows for the detection of most cases of MINS [14,19]. Systematic troponin testing should be considered as standard practice [10,12,18,21,22,25].

Therapeutic strategies remain limited. The efficacy of ASA, β-blockers, and ivabradine in reducing MINS has not been confirmed, and their use is often associated with increased complications [26,27,28]. Statins improve prognosis [8,10], while dabigatran is the only drug with documented efficacy in reducing major vascular events, as shown in the MANAGE trial [29]. In ENT, these findings are particularly relevant, as patients undergoing head and neck procedures frequently present with comorbidities, altered hemodynamics due to controlled hypotension, and a higher risk of silent MINS. Moreover, cardiac medications may interact with ENT-specific anesthetic techniques and perioperative bleeding risk. Multicenter trials focused on ENT populations are urgently needed to evaluate the safety and effectiveness of both pharmacological and non-pharmacological strategies.

Equally important is the close collaboration between ENT surgeons and anesthesiologists. Controlled hypotension, although essential for reducing bleeding and improving the surgical field, directly influences myocardial oxygen supply and perfusion. Effective teamwork is essential to balance surgical visibility with cardiovascular safety: the surgeon should clearly communicate the need for hypotension, while the anesthesiologist must carefully titrate its depth and duration, monitor troponin levels in high-risk patients, and promptly address hemodynamic instability. Such interdisciplinary cooperation allows for optimization of surgical conditions without compromising patient safety and may significantly reduce the risk of undiagnosed MINS [18,21,22,25]. Key Clinical Messages for Otorhinolaryngologists about MINS are shown in Table 2.

## 5. Conclusions

In summary, MINS remains an underestimated but significant complication in ENT surgery. Awareness of this problem among ENT specialists and anesthesiologists, routine monitoring of cardiac biomarkers, and further research into effective therapeutic strategies may contribute to improving patient safety in this group of operations.

## Figures and Tables

**Table 1:** Management strategies for MINS—summary of evidence.

Therapy	Evidence/Outcomes	Clinical Implications
Aspirin (ASA)	POISE-2 (>10,000 pts): no reduction in MI or death; ↑ major bleeding [26].	Routine perioperative ASA not recommended; decisions should be individualized.
β-blockers	POISE-1: ↓ MI (4.2% vs. 5.7%) but ↑ mortality and stroke [28].	Use with caution; potential harm outweighs benefits in MINS prevention.
Ivabradine	PREVENT-MINS (>2000 pts): ↓ HR but no reduction in MINS incidence [27].	Not effective for prevention.
Statins	Kashlan et al. (>2800 pts): ↓ cardiac complications by 23%; trend to ↓ 30-day mortality [10]. Intensive therapy ↓ 30-day mortality by 30–40% in MINS pts [8].	Statins recommended, especially high-intensity regimens (atorvastatin, rosuvastatin).
Dabigatran	MANAGE trial: ↓ major vascular complications by ~25–28% without ↑ major bleeding [29].	The only therapy with proven benefit in MINS; promising option.

Overview of available pharmacological strategies for the prevention and treatment of MINS, with results from major clinical trials and their implications for perioperative practice. **Abbreviations**: ASA—acetylsalicylic acid; MI—myocardial infarction; MINS—myocardial injury after non-cardiac surgery; pts—patients; ↑—increase; ↓—decrease.

**Table 2:** Key Clinical Messages for Otorhinolaryngologists About Myocardial Injury After Non-cardiac Surgery (MINS).

Aspect	Key Point
Incidence	MINS occurs in ~18% of patients after non-cardiac surgery; associated with 3–6× higher 30-day mortality and up to 4× higher 1-year mortality [11,12].
Clinical course	>80% of cases are asymptomatic; most would remain undetected without troponin testing [4,18].
Mechanism	Most commonly type II: imbalance between oxygen supply and demand, exacerbated by hypotension, tachycardia, anemia, hypoxemia [13,15,20].
ENT-specific risks	Controlled hypotension (MAP 50–65 mmHg) routinely used in FESS and middle ear microsurgery increases risk; every 10 min MAP < 55 mmHg raises MINS risk by ~30% [21,22,23,24].
Diagnostic gold standard	High-sensitivity troponins (hs-cTn), baseline and serial testing within the first 24–72 h postoperatively [2,14,19].
Practical implications	Limit depth/duration of hypotension; close ENT–anesthesia collaboration; consider troponin monitoring in patients ≥ 45 years or with CV comorbidities.

This table summarizes the most relevant aspects of MINS for clinical practice in otolaryngology, including incidence, clinical course, pathophysiological mechanisms, ENT-specific risks associated with controlled hypotension, diagnostic strategies, and practical recommendations. **Abbreviations**: MAP—mean arterial pressure; hs-cTn—high-sensitivity cardiac troponin; MINS—myocardial injury after non-cardiac surgery; ENT—ear, nose, and throat; CV—cardiovascular.

## Data Availability

The original data presented in the study are openly available in [PubMed], [Embase] and [Cochrane Library] at [pubmed.ncbi.nlm.nih.gov], [embase.com] and [cochranelibrary.com].

## Supplementary Materials

The following supporting information can be downloaded at https://www.mdpi.com/article/10.3390/jcm15062186/s1. PRISMA 2020 Checklist and PRISMA 2020 for Abstracts Checklist [30].

## Abbreviations

The following abbreviations are used in this manuscript:

MINS	Myocardial injury after non-cardiac surgery
MI	Myocardial infarction
FESS	Functional endoscopic sinus surgery
ENT	Ear, nose, and throat (otorhinolaryngology)
ECG	Electrocardiogram
hs-cTn	High-sensitivity cardiac troponins
NT-proBNP	N-terminal pro-B-type natriuretic peptide
MAP	Mean arterial pressure
HR	Heart rate
CI	Cardiac index
ICU	Intensive care unit
ASA	Acetylsalicylic acid
RR	Respiratory rate
Pts	Patients
CV	Cardiovascular
↑	Increase
↓	Decrease
