# Supplementary material for: Myocardial Injury After Non-Cardiac Surgery in Otolaryngology: Evidence Gaps and a Systematic Review
Source: J Clin Med. 2026 Mar 13;15(6):2186. doi: 10.3390/jcm15062186 (PMC13026464; doi:10.3390/jcm15062186)
Supplement: Supplementary file 1 [file jcm-15-02186-s001.zip › jcm-4177088PRISMA_2020_abstract_checklist MINS.pdf]

PRISMA 2020 for Abstracts Checklist

Section and Topic	Item #	Checklist item	Reported (Yes/No)
**TITLE**			
Title	1	Identify the report as a systematic review.	Lines 2-3
**BACKGROUND**			
Objectives	2	Provide an explicit statement of the main objective(s) or question(s) the review addresses.	Lines 17-20
**METHODS**			
Eligibility criteria	3	Specify the inclusion and exclusion criteria for the review.	Lines 22-23
Information sources	4	Specify the information sources (e.g. databases, registers) used to identify studies and the date when each was last searched.	Lines 21-22
Risk of bias	5	Specify the methods used to assess risk of bias in the included studies.	Not performed
Synthesis of results	6	Specify the methods used to present and synthesise results.	Line 23
**RESULTS**			
Included studies	7	Give the total number of included studies and participants and summarise relevant characteristics of studies.	Line 21
Synthesis of results	8	Present results for main outcomes, preferably indicating the number of included studies and participants for each. If meta-analysis was done, report the summary estimate and confidence/credible interval. If comparing groups, indicate the direction of the effect (i.e. which group is favoured).	Lines 21-31
**DISCUSSION**			
Limitations of evidence	9	Provide a brief summary of the limitations of the evidence included in the review (e.g. study risk of bias, inconsistency and imprecision).	Lines 28, 32-35
Interpretation	10	Provide a general interpretation of the results and important implications.	Lines 31-34
**OTHER**			
Funding	11	Specify the primary source of funding for the review.	This research received no external funding
Registration	12	Provide the register name and registration number.	Not registered

PRISMA

PRISMA 2020 for Abstracts Checklist

From: Page MJ, McKenzie JE, Bossuyt PM, Boutron I, Hoffmann TC, Mulrow CD, et al. The PRISMA 2020 statement: an updated guideline for reporting systematic reviews. BMJ 2021;372:n71. doi: 10.1136/bmj.n71. This work is licensed under CC BY 4.0. To view a copy of this license, visit https://creativecommons.org/licenses/by/4.0/
